# Workflow optimization of whole genome amplification and targeted panel sequencing for CTC mutation detection

**DOI:** 10.1038/s41525-017-0034-3

**Published:** 2017-11-01

**Authors:** Haiyan E. Liu, Melanie Triboulet, Amin Zia, Meghah Vuppalapaty, Evelyn Kidess-Sigal, John Coller, Vanita S. Natu, Vida Shokoohi, James Che, Corinne Renier, Natalie H. Chan, Violet R. Hanft, Stefanie S. Jeffrey, Elodie Sollier-Christen

**Affiliations:** 1 Vortex Biosciences, Inc., Menlo Park, CA USA; 2 Department of Surgery, Stanford University School of Medicine, Stanford, CA USA; 3 Stanford Center for Genomics and Personalized Medicine, Stanford University, Stanford, CA USA; 4 Department of Medicine, Division of Hepatology and Gastroenterology, Charité University Hospital, Berlin, Germany; 5 Stanford Functional Genomics Facility, Stanford University, Stanford, CA USA

## Abstract

Genomic characterization of circulating tumor cells (CTCs) may prove useful as a surrogate for conventional tissue biopsies. This is particularly important as studies have shown different mutational profiles between CTCs and ctDNA in some tumor subtypes. However, isolating rare CTCs from whole blood has significant hurdles. Very limited DNA quantities often can’t meet NGS requirements without whole genome amplification (WGA). Moreover, white blood cells (WBC) germline contamination may confound CTC somatic mutation analyses. Thus, a good CTC enrichment platform with an efficient WGA and NGS workflow are needed. Here, Vortex label-free CTC enrichment platform was used to capture CTCs. DNA extraction was optimized, WGA evaluated and targeted NGS tested. We used metastatic colorectal cancer (CRC) as the clinical target, HCT116 as the corresponding cell line, GenomePlex® and REPLI-g as the WGA methods, GeneRead DNAseq Human CRC Panel as the 38 gene panel. The workflow was further validated on metastatic CRC patient samples, assaying both tumor and CTCs. WBCs from the same patients were included to eliminate germline contaminations. The described workflow performed well on samples with sufficient DNA, but showed bias for rare cells with limited DNA input. REPLI-g provided an unbiased amplification on fresh rare cells, enabling an accurate variant calling using the targeted NGS. Somatic variants were detected in patient CTCs and not found in age matched healthy donors. This demonstrates the feasibility of a simple workflow for clinically relevant monitoring of tumor genetics in real time and over the course of a patient’s therapy using CTCs.

## Liquid biopsy: Simple workflow allows DNA analysis of circulating tumor cells

A microfluidic device that isolates cancer cells circulating in a blood sample allows for real-time genetic monitoring. A team led by Elodie Sollier-Christen of Vortex Biosciences, a cancer diagnostics company in Menlo Park, California, USA, in collaboration with Professor Stefanie Jeffrey at Stanford University School of Medicine, developed a simple workflow for analyzing the genomes of rare circulating tumor cells (CTCs) found in the bloodstream after they’ve been collected through a proprietary microfluidic system. They optimized rare cell DNA extraction, compared different whole genome amplification methods, and then tested the workflow on blood samples from patients with metastatic colorectal cancer. The analysis also included white blood cells from the same patients to parse cancer-causing mutations from inherited ones. The method could aid in the translation of liquid biopsies for the clinical care of cancer patients.

## Introduction

Circulating tumor cells (CTCs) are cancer cells shed into the blood stream by both primary and metastatic tumors. Their importance as prognostic biomarkers has been well demonstrated, and CTC characterization is now playing a growing role in the era of personalized medicine.^1–3^ Traditional tumor tissue biopsies may be painful, risky, expensive, and limited by the difficulty of accessing the tumor site. Furthermore, single-site tumor biopsies may not recapitulate intra-tumor and inter-tumor heterogeneity, particularly if multiple metastases are present, and may fail to reflect the genetic diversity of a patient’s disease. These limitations can be overcome with non-invasive blood tests, called liquid biopsies, and importantly include the isolation and analyses of CTCs. Liquid biopsy facilitates serial sampling to enable real-time and more accurate monitoring of disease during tumor evolution and through assessment of patient response to treatment changes, ultimately providing a more personalized and time-sensitive treatment of the cancer. Previous studies have shown that CTC enumeration and mutational profiling may be used to monitor cancer disease,^4^ and to predict progression and overall survival of cancer patients.^5–7^ This is particularly important because several studies have suggested that in some tumor subtypes, such as colorectal or lung cancer, some patients’ CTCs and cell-free ctDNA may show different mutational profiles.^8–11^ However, isolating rare CTCs from millions of white blood cells (WBCs) and billions of red blood cells (RBCs) has significant hurdles. Affinity-based technologies, such as CellSearch,^12,13^ rely on molecular biomarkers like epithelial cell adhesion molecule (EpCAM) to be expressed on the surface of CTCs. Some cancer types and their CTCs, however, may not express EpCAM.^14^ Also, CTCs are capable of transitioning from an epithelial to mesenchymal phenotype, rendering the cells more aggressive and invasive.^15–19^ Along this transition, CTCs down-regulate EpCAM, which implies that an affinity-based capture method may miss the most clinically relevant and aggressive CTCs. Size-based filtration methods overcome this issue through capture of a more diverse population of CTCs, but may require prior fixation of the cells^20^ or pressures on the cells during the filtration procedure that may potentially affect downstream assays. The Vortex technology^21,22^ is a label-free microfluidic chip that relies on laminar microvortices to isolate and concentrate CTCs from blood, based solely on their physical properties such as size and deformability. As published previously, our technology enables a rapid CTC enrichment at high purity, while enabling the collection of intact CTCs in an Eppendorf tube, well-plate (strip), or other collection tube, depending on the downstream analytical assay. No transfer of the sample is required, and CTCs are directly available for immunofluorescence or genomic assays.

Genomic mutational profiling of CTCs provides pertinent information for personalized therapy.^23^ Targeted next-generation sequencing (NGS) allows rapid detection of a variety of mutations of a gene panel on single platforms (such as Ion Torrent and MiSeq).^24,25^ Targeted NGS is especially useful to guide treatment when focusing on a drug actionable gene panel, with a fast turnaround time and a low cost, such as looking for mutations of *KRAS, EGFR,* or *ALK/ROS1* rearrangements to guide the selection of cetuximab, erlotinib/Gefitinib, or crizotinib for cancer patients,^26,27^ respectively. Compared to whole-genome sequencing, targeted NGS also allows the identification of rare variants to high depth but with a smaller and consequently more manageable data set, making data analysis much easier and faster.

Using targeted NGS on CTCs would unleash the potential of CTCs as a surrogate for conventional biopsies.^28^ However, there are several challenges: (i) *CTCs are extremely rare* in the blood, with a usual range of 1–100 CTCs per ml of whole blood.^29,30^ Given that a single diploid cell contains only 6–7 pg of DNA, the amount of DNA obtained from even hundreds of CTCs would still be well below the input required as most of the current NGS platforms require ng or µg of DNA. Thus, whole-genome amplification (WGA) is required prior to NGS. Several strategies for WGA have been developed, such as REPLI-g technology (Qiagen) that uses multiple displacement amplification (MDA), and GenomePlex approach (Sigma-Aldrich) that uses PCR to amplify the entire genome.^31^ Both strategies are broadly used, yielding different performances and biases, and allowing for different specific applications. These two methods were compared in this study for their use on CTCs. (ii) *Low Purity of CTCs*. CTCs are among millions of WBCs and billions of RBCs. The molecular characterization of isolated CTCs is very challenging as the number of simultaneously isolated WBCs can vary from hundreds to tens of thousands depending on the CTC enrichment platforms, which is especially problematic for NGS. Moreover, contaminating WBCs will carry germline mutations that may be confused with somatic mutations from the CTCs. Thus, WBC germline control might be needed to eliminate potential background if using pooled cells. (iii) *CTCs are collected in varying conditions and from different platforms*. CTCs can be collected in a microtube or a well-plate, and they can be freshly frozen or fixed. Being able to use a similar NGS workflow on both fresh and fixed cells, whatever their collection container, would be highly desirable and would enable both enumeration and NGS on the same CTC sample. However, fixatives might damage DNA by inducing nucleic acid cross-linking^32^ and may not be compatible with WGA approaches, increasing WGA error rates.^33^ Evaluation of fixed cells is thus also needed to answer this question.

**Fig. 1:**
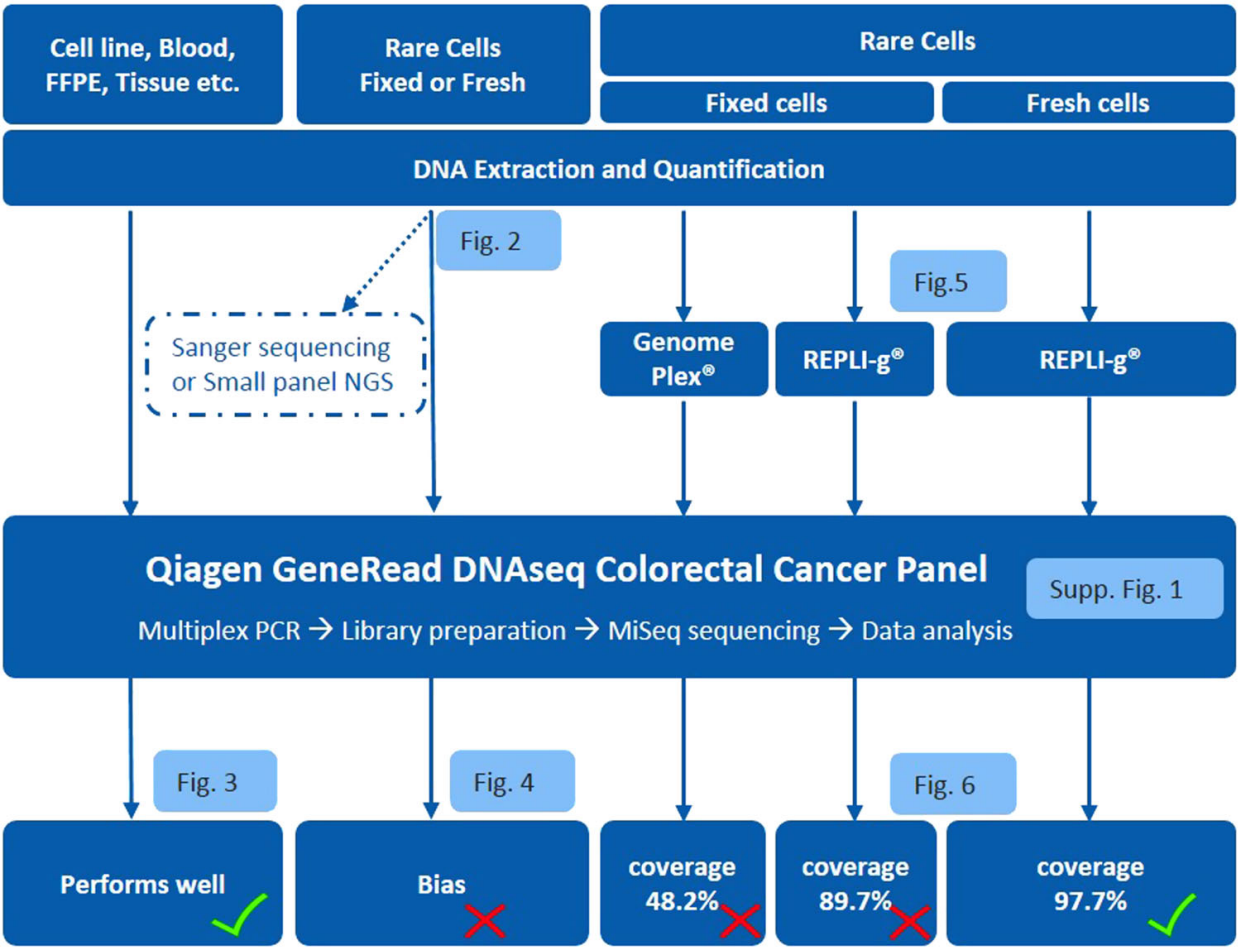
Workflow optimization for biopsy genomic profiling, tissue and CTCs. Qiagen QIAamp Micro Kit and GeneRead DNAseq targeted panel sequencing were applied on all types of cell samples. This overall workflow performs well when DNA amount is sufficient, i.e., from whole blood and tissue biopsy. For rare cells, DNA input is insufficient for targeted NGS, leading to a bias and the need for an extra step of Whole Genome Amplification (WGA). Two WGA kits were evaluated (WGA4 from Sigma and REPLI-g from Qiagen) and obtained low coverage for fixed cells, while REPLI-g was identified as optimal for fresh rare cells and used for a final validation on patient CTCs

In this study, we tested different workflows starting from the optimization of DNA extraction to WGA evaluation and then targeted MiSeq NGS, to determine the optimal methods for genomic analysis of tissue biopsies, formalin-fixed paraffin-embedded (FFPE) samples, fresh or fixed cells and rare CTCs (Fig. 1). To test and optimize this genomic workflow, we chose metastatic colorectal cancer as the clinical target, HCT116 as the corresponding cell line, and the GeneRead DNAseq targeted Human Colorectal Cancer (CRC) Panel V2 (Qiagen) as the gene panel. The overall workflow was further tested on metastatic CRC patient samples, assaying both tumor biopsies and CTCs isolated from blood samples by Vortex technology. Ultimately, this study reveals the feasibility of a simple workflow for clinically relevant monitoring of tumor genetics in real time and over the course of a patient’s therapy by analyzing CTCs.

## Results

### DNA extraction optimization for fresh and fixed cells

#### Comparison of different commercial kits

To test different DNA extraction kits, HCT116 cells were collected freshly or after fixation with 4% PFA, in a 1.5 mL micro-centrifuge tube or in a 96 well-plate, with 200–300 cells per sample. DNA extraction was performed simultaneously from different aliquots of the same cell suspension, using the following kits, respectively: QIAamp DNA Micro kit (Qiagen), GeneRead DNA FFPE kit (Qiagen), ZR Genomic DNA™ Tissue MicroPrep kit (Zymo) and Arcturus® PicoPure® DNA Extraction kit (Life technologies). For each kit and protocol considered, both DNA yield and user experience were recapitulated in Table 1.

**Table 1:** Comparison of different commercial kits for DNA extraction from rare cells

Cells	Kit	Protocol	1.5 ml tube	96 well-plate	Comments
HCT116 Fresh	Qiagen QIAamp DNA Micro	Cell Protocol	6–7 pg/cell (~100%)	6-7pg/cell (~100%)	• Cell protocol performs well for a small amount of fresh cells, both in a tube or in a well-plate.
					• Recommended for small amount of fresh/frozen cells.
	Qiagen QIAamp DNA Micro	Cell Protocol	0.1–0.5 pg/cell (<1%)	Not Tested	• Not optimal for fixed cells.
		Tissue Protocol	3–4 pg/cell (~50%)	3–4 pg/cell (~50%)	• Tissue protocol performs well for a small amount of fixed cells.
					• Selected for further optimization on fixed, permeabilized, immuno-stained cells.
HCT116 Fixed	Qiagen GeneRead DNA FFPE	Standard Protocol	2–3 pg/cell (~30%)	Not Tested	• Not optimal for fixed cells.
					• Procedure is more complicated, with hazardous chemicals involved.
	Zymo ZR Genomic DNA™ Tissue MicroPrep	Standard Protocol	3–4 pg/cell (~50%)	3–4 pg/cell (~50%)	• Could be used as a substitute for Qiagen Micro Kit.
					• No further optimization has been performed.
	Life technologies Arcturus® PicoPure® DNA Extraction	Standard Protocol	3–4 pg/cell (~50%)	0.5–1 pg/cell (<10%)	• Simple workflow with Applied Biosystems® Arcturus® LCM instruments.
					• Small digestion volume causes evaporation during the incubation when using a 96 well-plate.

HCT116 cells were prepared fresh, or fixed with 4% PFA, collected in a 1.5 mL micro-centrifuge tube or in a 96 well-plate (~200–300 cells/experiment, *N* ≥ 2 per condition). DNA extraction kits tested side-by-side included kits from: Qiagen, Zymo and Life Technologies. One hundred percent DNA recovery was defined assuming a DNA yield of about 6 pg per fresh diploid cell. Qiagen QIAamp DNA Micro Kit was selected for both tissue biopsy and for further optimization on CTC-like samples

Similar good results were achieved for fresh HCT116 cells seeded in a micro-centrifuge tubes and in well-plates using the Qiagen QIAamp DNA Micro kit with the manufacturer’s standard cell protocol, with a DNA yield of 6–7 pg/cell, similar to the expected yield. This yield was set at 100% as a reference. When the QIAamp DNA Micro kit and standard cell protocol were tested on HCT116 cells fixed with 4% PFA, DNA yield was as low as 0.1–0.5 pg/cell, i.e., around 1% of the yield obtained for fresh cells (Table 1). DNA yield increased to 3–4 pg/cell (~50% of the DNA from fresh cells) in both micro-centrifuge tube and well-plate formats by using the same kit but with the standard tissue protocol. Other kits showed similar performance, with a DNA yield of 2–3 pg/cell (~30% recovery) for the GeneRead DNA FFPE kit, and 3–4 pg/cell (~50% recovery) for both ZR Genomic DNA™ Tissue MicroPrep kit and Arcturus® PicoPure® DNA Extraction kit. Among these kits, the QIAamp DNA Micro kit was selected for further in-house optimization towards the increase of DNA recovery for fixed cells because this kit can be used for both fixed and fresh cells, with simple procedures and less hazardous chemicals involved (Table 1).

#### Optimization of DNA extraction from rare and fixed cells

**Fig. 2:**
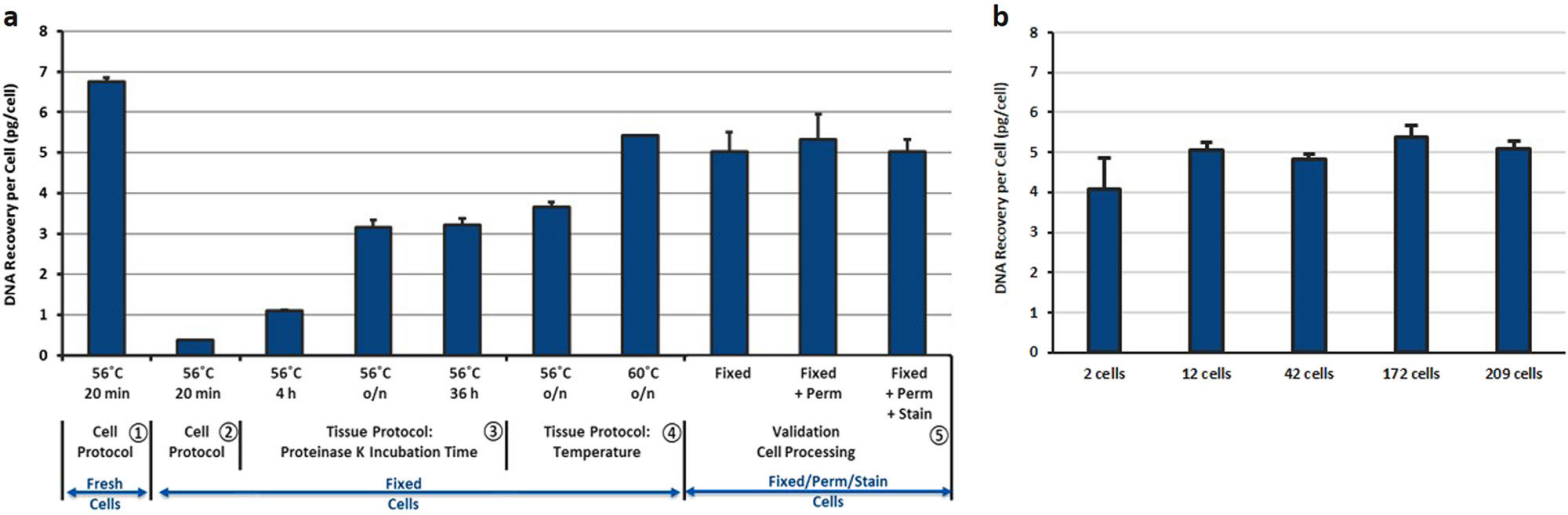
**a** Optimization of DNA extraction from rare and fixed cells using Qiagen QIAamp DNA Micro Kit. ~200–300 cells/experiment, *N* ≥ 2 per condition. ① Fresh cells using Cell Protocol yielded 6–7 pg of DNA/cell and were used as a control. ② Cell Protocol did not work on cells fixed with 4% PFA. ③ Tissue Protocol significantly increased the DNA yield when Proteinase K digestion was extended from 4 to 36 h, with significant advantage of overnight incubation. ④ Increasing the Proteinase K digestion temperature from 56 to 60 °C further increased the DNA yield from 60 to 80%. ⑤ The optimized protocol was then verified on fixed, fixed+permeabilized and fixed+permeabilized+stained HCT116 cells to mimic the output from CTC enrichment platforms. **b** Validation of an optimal DNA extraction protocol for low number of fixed cells. Different amounts of HCT116 cells fixed with 4% PFA were seeded inside a 96 well-plate, imaged and counted. DNA was extracted from these cells using the optimized protocol defined in A (Qiagen QIAamp DNA Micro Kit, Tissue Protocol, Proteinase K digestion overnight at 60 °C) and quantitated using qPCR

To increase DNA recovery, different Proteinase K incubation times (20 min, 4 h, overnight, 36 h) and incubation temperatures (56 °C, 60 °C) were tested (Fig. 2a). An extended incubation time of Proteinase K from the initial 4 h to overnight increased the DNA yield from 20–30% to 50–60%, while prolonging for up to 36 h did not bring additional improvement. Increasing incubation temperature to 60 °C instead of 56 °C combined with overnight Proteinase K digestion further increased the DNA yield from 60 to 80%.

#### Validation of the optimized DNA extraction method for rare cells

To mimic real outputs from various CTC enrichment platforms, the optimized DNA extraction workflow was applied on cells that had been through fixation, permeabilization and immunostaining, and provided a DNA recovery of 80%. To test the compatibility of the workflow on rare cells, a low number of cells was processed (Fig. 2b) and a constant DNA yield of 80% was obtained for a cell number varying from 2 to 200 cells.

### Validation of targeted panel next generation sequencing

#### When DNA amount is higher than 40 ng

**Fig. 3:**
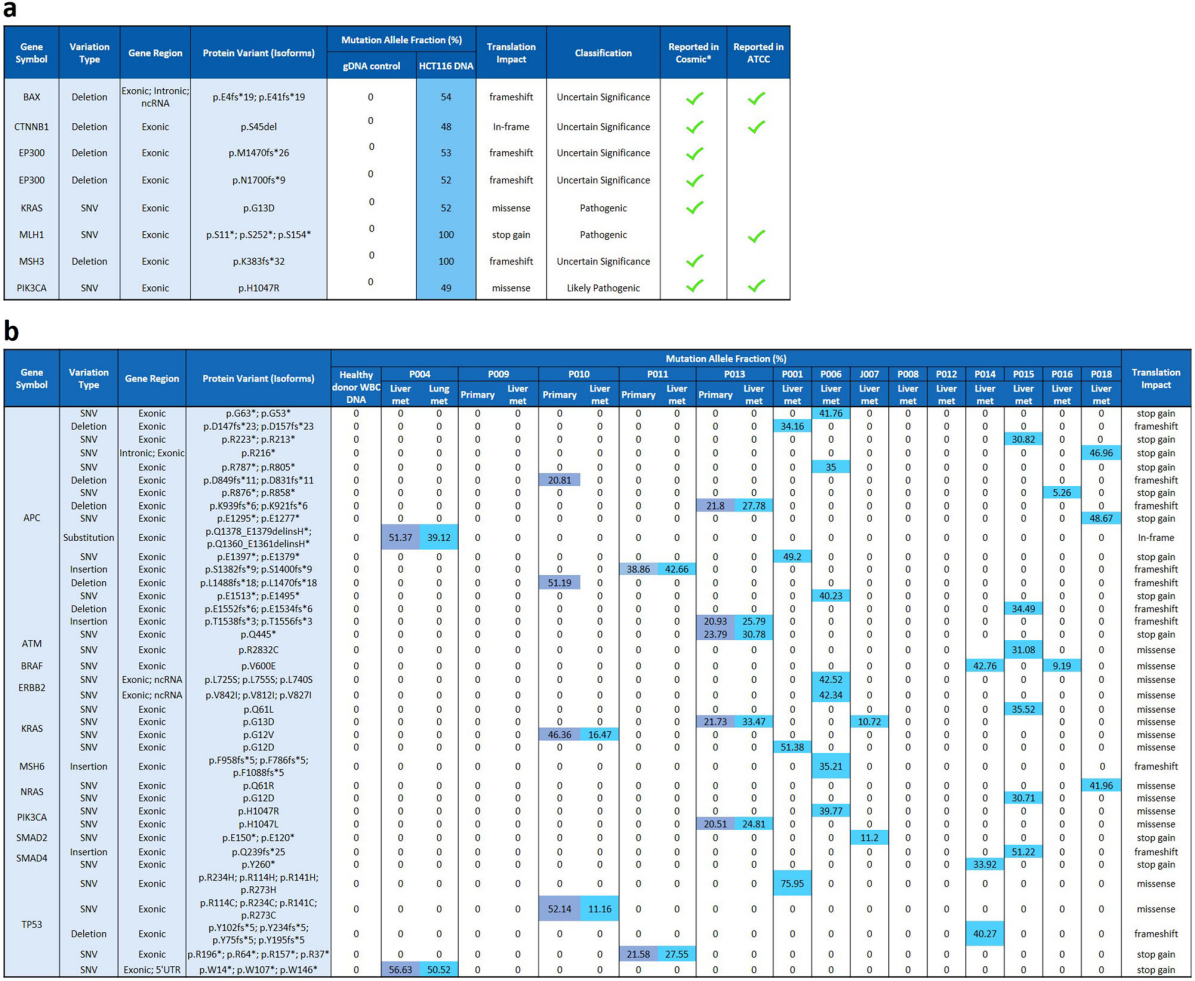
Mutation detection using GeneRead DNAseq targeted CRC panel sequencing. **a** Mutation detection in HCT116 cancer cells. Forty nanogram of DNA extracted from fresh HCT116 cells were subjected to multiplex PCR, library preparation and MiSeq sequencing. Mutations and corresponding allele frequencies were called using Ingenuity variant analysis software and compared to results reported from Cosmic* (catalog of somatic mutations in cancer) and ATCC websites. **b** Mutation detection in CRC patient biopsy tissues. Forty nanogram of DNA extracted from primary tumor and liver metastasis were subjected to multiplex PCR, library preparation and MiSeq sequencing. Mutations and corresponding Mutation Allele Fraction (MAF) were analyzed and compared between primary tumor and liver metastasis. In both tables, the blue color highlights the detected mutation and the number inside each cell represents the MAF of the mutation

The whole workflow, including Qiagen GeneRead DNAseq CRC Targeted Panel v2 (NGHS-002X, Qiagen) and MiSeq NGS, was tested on HCT116 DNA, human genomic DNA and tumor tissue DNA with a DNA input of 40 ng as recommended (Fig. 1, Supplementary Figure 1, Fig. 3). When applied on 40 ng of DNA from fresh HCT116 cells (Fig. 3a), eight variants were detected, including *KRAS* (p.G13D), *PIK3CA* (p.H1047R), and *CTNNB1 (*p.S45del), with all of them being reported in ATCC or in COSMIC (Catalog of Somatic Mutations In Cancer). As expected, the mutation allele fraction (MAF) was 100% for homozygous mutations such as *MLH1* (p.S11*; p.S252*; p.S154*) and *MSH3* (p.K383fs*32), and around 50% for the heterozygous mutations such as *BAX* (p.E4fs*19, p.E41fs*19), *CTNNB1* (p.S45del), *EP300* (p.M1470fs*26, p.N1700fs*9), *KRAS* (p.G13D), *PIK3CA* (p.H1047R), which confirms the good coverage of the workflow. No mutation was detected in the Roche human blood genomic control DNA as expected.

This workflow was then applied on tissue specimens including three primary tumor FFPE samples, 14 fresh frozen liver metastatic tumor samples and one lung metastatic tumor FFPE sample collected from 14 CRC patients (Fig. 3b). Roche genomic DNA was included as control, with no mutation detected. In 11 of 14 liver metastases (78.6%) at least two mutations were detectable. Four of the patients had both liver metastases and FFPE primary (3) or lung metastatic (1) tissue available: 100% concordance was obtained between the primary tumor and the liver metastasis of patients P011 and P013, and between the liver and lung metastatic tissue of patient P004. For the patient P010, two more APC mutations were detected in primary tumor than in the liver metastasis. This further demonstrated the good performance of the overall workflow for analysis of tumor tissue and FFPE sections.

#### When DNA amount is lower than 40 ng

**Fig. 4:**
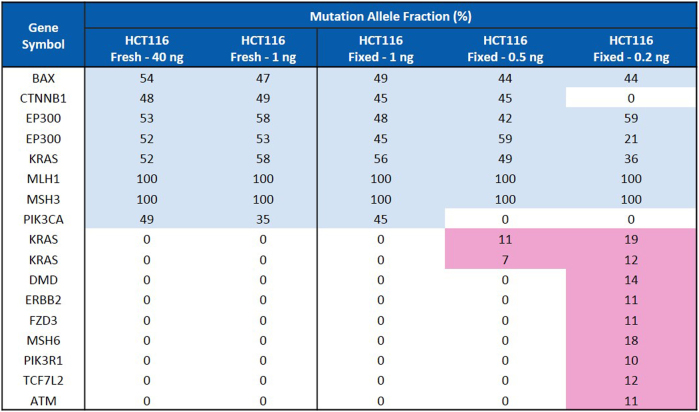
Mutation detection in HCT116 cancer cells with different DNA input using GeneRead DNAseq targeted CRC Panel. Forty and 1 ng DNA extracted from fresh HCT116 cells were compared to 1.0, 0.5, and 0.2 ng of DNA extracted from HCT116 cells fixed with 4% PFA. In all conditions, DNA was subjected to multiplex PCR, library preparation and MiSeq sequencing. Mutations and corresponding allele frequencies were analyzed and compared

The workflow was then applied on a lower amount of DNA from HCT116 cells, including 1.0, 0.5, and 0.2 ng (Fig. 4). When DNA amount was reduced to 1.0 ng, the workflow still performed well: the mutations detected from either fresh or fixed HCT116 cells were same to 40 ng of DNA. Eight mutations were detected with similar allele frequencies among these three samples (Fig. 4). This indicates the compatibility of this workflow with fixed cells and a DNA amount as low as 1.0 ng without introducing obvious bias. However, when using DNA amounts of 0.5 and 0.2 ng from fixed cells, an inaccuracy in the results was observed: Some mutations were missed while some false positive mutations were called (in light red). This highlights the need for an extra step of WGA to be able to perform targeted NGS on rare cells (DNA amount inferior to 1 ng).

### WGA on fresh and fixed cells

**Fig. 5:**
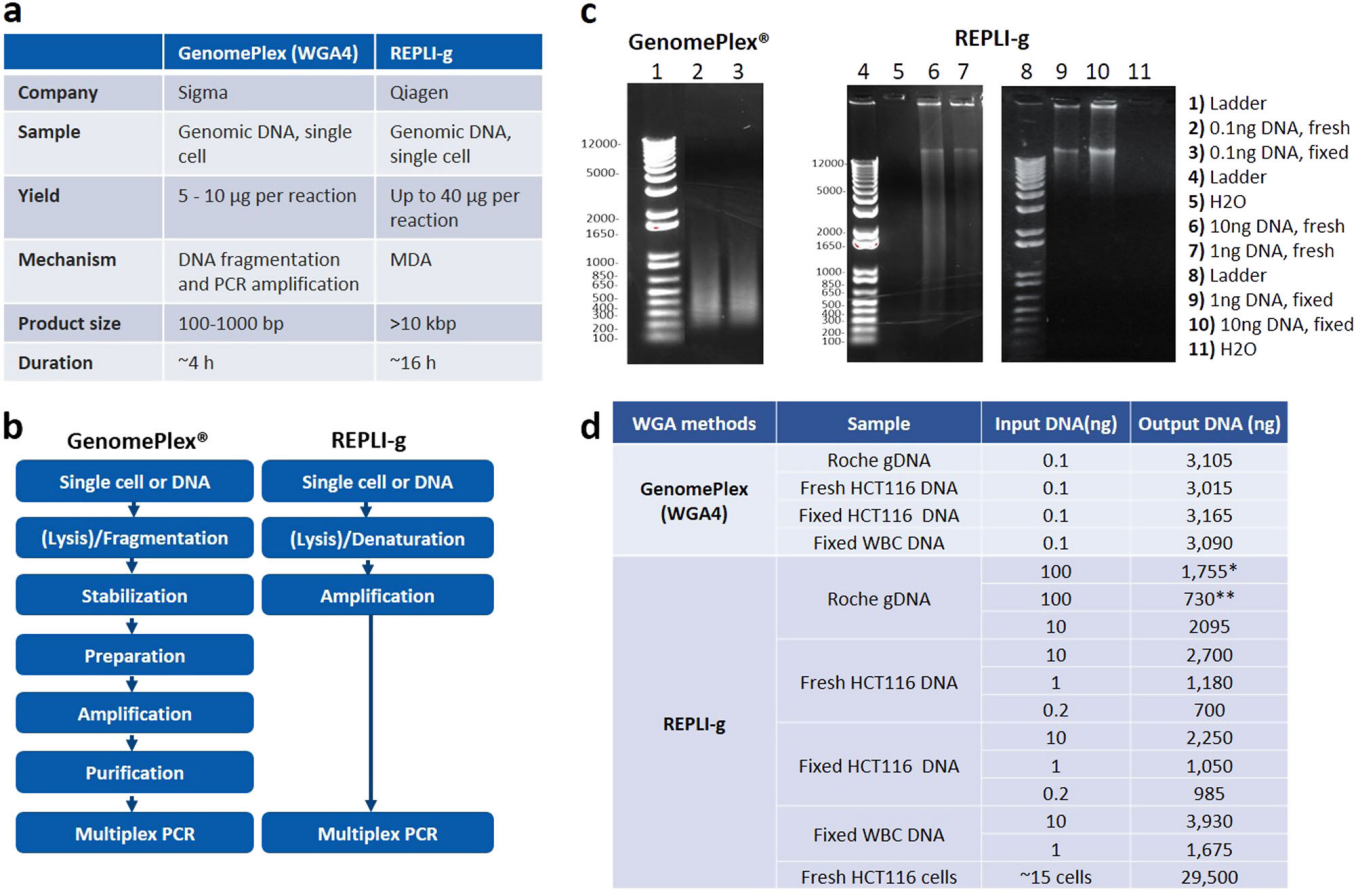
Whole genome amplification by using GenomePlex (WGA4) and REPLI-g. **a** Comparison of two whole genome amplification methods: GenomePlex (WGA4) and REPLI-g. **b** Workflow of GenomePlex (WGA4) and REPLI-g kits. **c** Whole Genome Amplified DNA products. GenomePlex (WGA4) or REPLI-g WGA was performed on DNA from both fresh and fixed HCT116 cells following the vendor’s manuals. The amplified DNA was checked using agarose gel electrophoresis. **d** DNA yield comparison. GenomePlex (WGA4) or REPLI-g WGA was performed on both fresh and fixed HCT116 cells following the vendor’s manuals. The amplified DNA amount was measured using the Qubit. (*: standard protocol. **: increased DNA volume protocol)

WGA was performed using GenomePlex® Single Cell Whole Genome Amplification Kit (WGA4) and REPLI-g® Single Cell Kit. The major differences between these two kits are listed in Figs. 5a, b. The quality of the WGA DNA was determined by agarose gel electrophoresis (Fig. 5c) and the DNA yield was measured by Qubit (Fig. 5d).

0.1 ng of Roche human genomic DNA, fresh HCT116 DNA and fixed HCT116 DNA were amplified using GenomePlex kit, yielding around 3000 ng of DNA for all three samples. The amplified DNA fragments range from 100 to 1000 bp, with a mean size of around 300–500 bp (Fig. 5c), similar to the manufacturer’s expectations. The water control was not amplified.

In parallel, 100 ng of Roche human gDNA was amplified using REPLI-g kit following either the manufacturer’s “standard protocol” (starting DNA volume of 2.5 µl) or the “increased DNA volume protocol” (starting DNA volume of 15 µl). In both protocols, the step for isothermal amplification at 30 °C for 8 h was performed in the PCR thermocycler with the lid temperature set at 70 °C as suggested. The DNA yield from these amplifications was 1755 ng and 730 ng, respectively, for the two protocols (Fig. 5d). By adjusting the lid temperature to 50 °C (i.e., the lowest temperature that can be set in the thermocycler), the DNA yield reached 2095 ng from a starting input of only 10 ng gDNA. Thus, this lid temperature was selected for the following experiments. Different amounts of fresh and fixed HCT116 DNA and WBC DNA were amplified (Fig. 5d). The DNA yield from fixed HCT116 DNA was generally slightly lower than fresh HCT116 DNA. As a validation of the workflow directly on cells, (~15 fresh HCT116 cells were lysed and their DNA amplified, with a DNA yield of about 30 µg (Fig. 5d). The main REPLI-g amplified DNA fragments were longer than 12 kb, in accordance with the product length indicated in the handbook (>10 kb) (Fig. 5c), while the water control was not amplified. The DNA amplified using both kits was subjected to NGS to further verify the coverage and WGA performance.

### Targeted NGS on WGA-amplified DNA from both fresh and fixed HCT116 cells

**Fig. 6:**
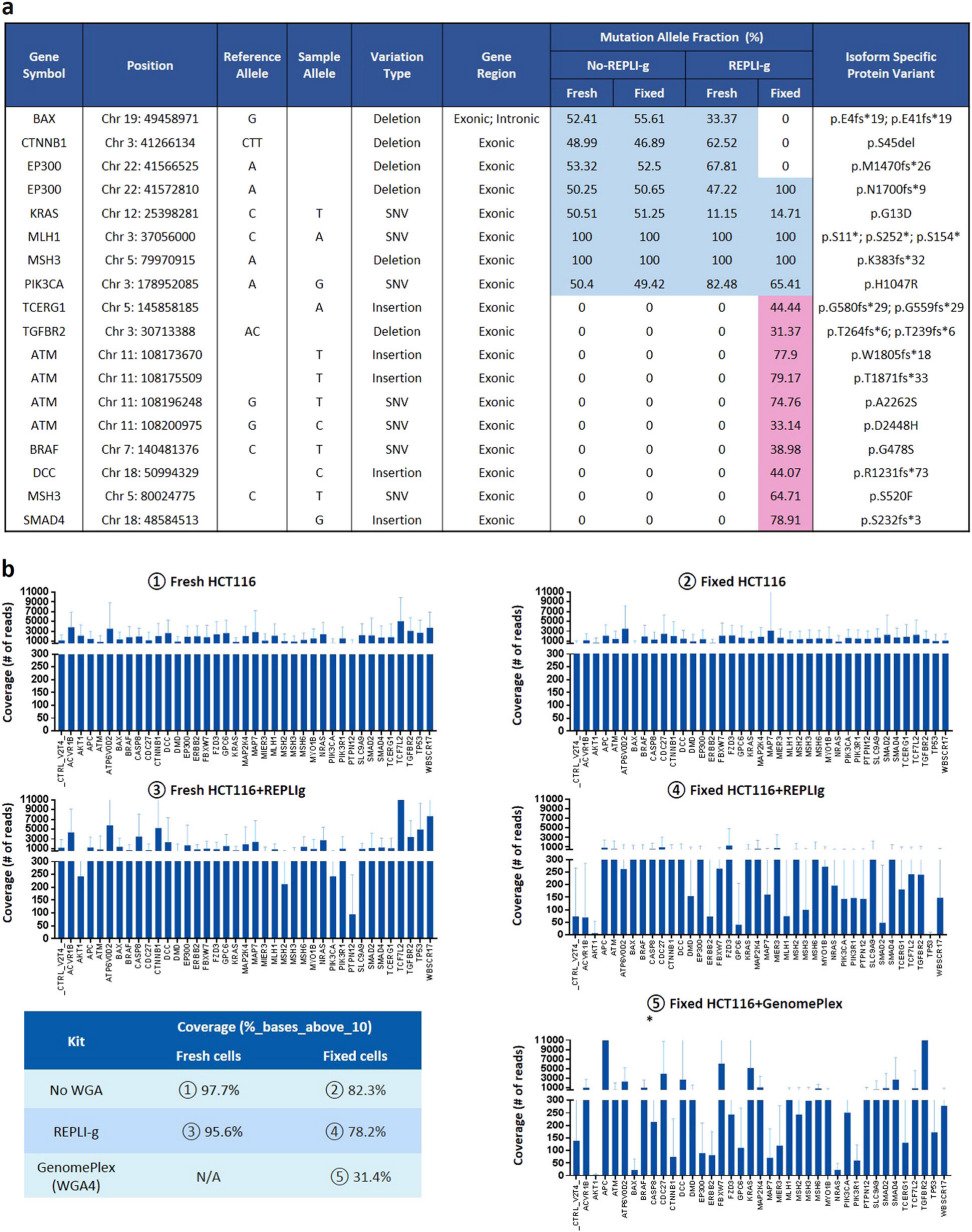
Sequencing comparison in WGA amplified and non-amplified DNA samples. **a** Mutation detection comparison for REPLI-g WGA. REPLI-g amplified or non-amplified DNA from both fresh and fixed HCT116 cells were subjected to the CRC targeted NGS. The blue color highlights the true mutations detected, while the number inside each cell represents the MAF of the mutation. The light red color highlights the false mutations called. **b** Gene coverage comparison for WGA. The coverage was compared among the following samples: ① Fresh cells; ② Fixed cells; ③ Fresh cells + REPLI-g; ④ Fixed cells + REPLI-g. ⑤ Fixed cells+WGA4. The reads were aligned using BWA-MEM and coverage was computed using GATK’s DepthOfCoverage and in-house scripts. The percentage of bases covered by at least 10 reads with minimum base quality score Q30 (%_bases_above_10) was calculated using data obtained from DepthOfCoverage and in-house scripts and summarized in the table. (*) In the case of GenomePlex (WGA4), the plotted data was trimmed of 10 bp at the beginning and 40 bp at the end because of a primer/adapter contamination

Forty nanogram of amplified or non-amplified DNA from fixed or fresh HCT116 cells using GenomePlex (WGA4) or REPLI-g kits was subjected to targeted NGS to evaluate the performance of each kit. For GenomePlex (WGA4) amplified DNA, too much primer/adapter contamination was observed in reads and the final mutations were not successfully detected. For REPLI-g amplified DNA, the NGS performed well as highlighted with the detected mutations and the corresponding MAF listed in Fig. 6a.

For fresh HCT116 cells, both REPLI-g amplified DNA and non-amplified DNA called for the same eight mutations with a similar MAF for several of these mutations (highlighted in blue). No extra mutation was detected in REPLI-g amplified DNA. For example, the MAF of two homozygous mutations MLH1 (p.S11*; p.S252*; p.S154*) and MSH3 (p.K383fs*32) were 100% in both samples. EP300 (p.N1700fs*9) had 47.22% in amplified sample and 50.25% in non-amplified sample. Other mutations showed different allele frequencies, such as KRAS (p.G13D) (11.15% vs. 50.51%) and PIK3CA (p.H1047R) (82.48% vs. 50.4%).

For fixed cells, three mutations were not caught in REPLI-g amplified DNA: BAX (p.E4fs*19 p.E41fs*19), CTNNB1 (p.S45del) and EP300 (p.M1470fs*26), while ten extra mutations (highlighted in red) including ATM, BRAF, DCC, and SMAD4 were falsely detected (Fig. 6a).

To further understand the gene distribution and coverages, and compare the two WGA kits, the percentage of bases covered by at least 10 reads with minimum base quality score Q30(%_bases_above_10), was calculated and reported using DepthOfCoverage in the Fig. 6b. In non-amplified DNA samples (① and ②), the coverage of fresh and fixed cells was 97.7 and 82.3%, respectively. In REPLI-g amplified DNA samples, the coverage was 95.6% for the fresh cells ③ but dropped to 78.2% for the fixed cells ④. The gene coverage of the GenomePlex (WGA4) amplified DNA was only 31.4%% ⑤, which might be due to the primer/adapter contamination in the reads. To understand further the drop in coverage for fixed cells with REPLI-g, the MAF of mutations between REPLI-g amplified and non-amplified samples were compared (Supplementary Figure 2). In both fresh and fixed non-amplified samples, the allele frequencies of SNPs detected mostly remain around 100 and 50%. However, in REPLI-g amplified samples, the allele frequencies were scattered and did not show any trends. This explains the MAF difference observed in Fig. 6a and may be caused by allelic dropout (and consequent strand-bias) associated with WGA methods that are based on MDA. The allelic dropout in WGA has been studied previously.^34^

### Enumeration and mutational profiling of patient CTC samples

**Fig. 7:**
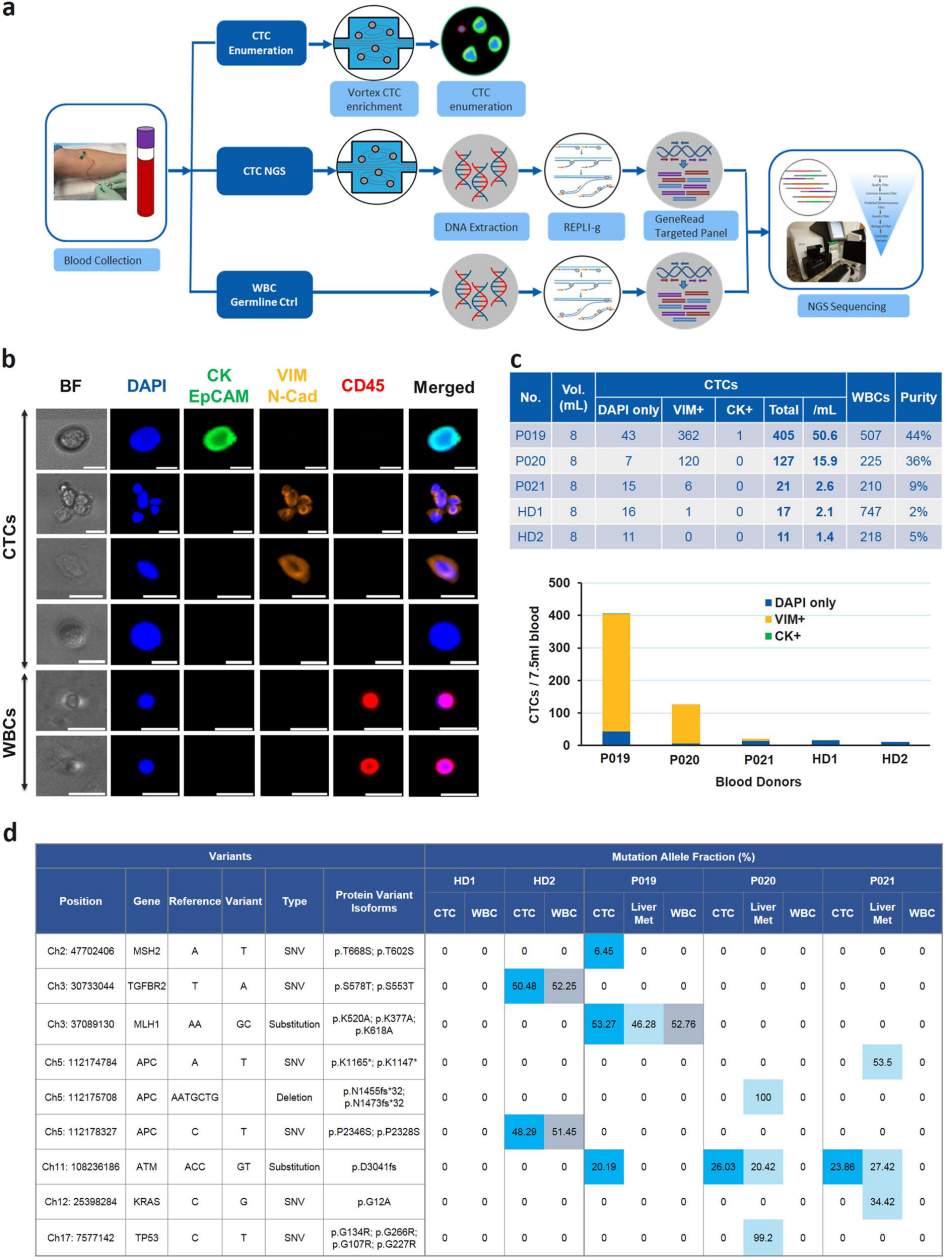
Enumeration and mutational profiling of patient CTC samples. **a** Workflow summary. Blood samples were collected into two EDTA tubes from three CRC patients with resectable hepatic metastases and two age-matched healthy donors. One tube of blood (8 ml) was processed through Vortex technology to collect CTCs for fixation, immunostaining and enumeration. The other tube (8 mL) was also processed through Vortex to collect CTCs for DNA extraction, WGA, PCR, library preparation and MiSeq sequencing. 100 µl of whole blood collected from all patients and healthy donors was stored as the germline control and processed through the same genomic workflow. The sequencing results obtained from the whole blood were compared to the ones of the CTC samples and subtracted as the background. **b** Picture gallery. Pictures of CTCs and WBCs immunostained with CK, EpCAM, Vimentin, N-Cadherin, CD45, and DAPI. Scale bar represents 20 µm. **c** CTC enumeration results of each CRC patient and age-matched healthy donor. **d** Mutation detection in CTCs, liver metastases and WBCs. In all conditions, DNA was extracted and subjected to targeted NGS. The blue/light blue/gray colors highlight the mutations detected in CTCs/liver metastatic tissue/WBCs, respectively, while the number inside each cell indicates the MAF of the mutation. Note that we are calling a genetic variant a SNP when it is also found in corresponding WBCs from the same patient, as in MLH1 in case P019, stressing the importance of including the WBCs as germline controls for each patient

The final workflow was validated on three metastatic CRC patients (study II: P019, P020, P021) and two healthy donors (HD1, HD2) (Supplementary Table 1). For each patient and healthy donor, two tubes of blood were collected. Both tubes were processed through Vortex microfluidic device to collect the CTCs, and the CTCs from one tube were stained and enumerated while the CTCs isolated from the second one were used freshly for mutation profiling (Fig. 7a). The CTC enumeration and mutation results are summarized in Figs. 7 b, c.

Respectively 50.6, 15.9, and 2.6 CTCs per mL of blood were collected for patients P019, P020, and P021 with a contamination of 63, 28, 26 WBCs/mL (i.e., 9, 36, and 44% purity). More cells were collected from the three patient samples than in the two age-matched healthy donors: 16 and 11 of CTC-like cells per tube in donors HD1 and HD2 respectively. Most of the patient CTCs were Vimentin and N-Cadherin positive, with for example 89.4 and 94.5% of VIM/N-Cad+ CTCs in patient P019 and P020. Besides, in these two patients, many VIM/N-Cad+ CTC clusters were also observed (Fig. 7b). For the two healthy donors, 94 and 100% of the CTC-like cells were VIM/N-Cad- and CK-.

The mutation detection on collected cells (a mixture of CTCs and WBCs) showed that, no somatic mutation was found in the healthy donors. Two germline mutations: TGFBR2 (Ch3: 30733044, T>A) and APC (Chr 5:112178327, C>T) were detected in both WBCs and pooled CTC samples from HD2. This emphasizes the importance of WBC germline control if using pooled CTC samples for mutation profiling. In P019, 3 mutations: MLH1 (Chr3: 37089130, delAAinsGC), MSH2 (Chr 2: 47702406, A>T) and ATM (Chr11:108236186, delACCinsGT) were detected in CTC samples, with among them MLH1 being present in both liver metastases and WBC control. WBC germline controls are important, as illustrated here, where the MLH1 variant found in all three compartments (WBCs, liver metastases, and CTCs) is most likely a single nucleotide polymorphism (SNP) rather than a mutation. This indicates that only MSH2 and ATM were real mutations in CTCs. In P020, no mutation was detected in the WBC control, whereas ATM (Chr11:108236186, delACCinsGT) was present in CTCs, and ATM, APC (Chr5:112175708, delAATGCTG), TP53 (Chr17:7577142, C>T) present in liver tissue. This suggests that more mutations were detected in CTCs than in the tumor for P019, whereas for P020 it was the opposite, namely less mutations were detected in CTCs than in the tumor. P021 showed similar results as P020: one mutation was detected in CTCs and two additional mutations in tumor tissue. A total of only three patients including blood and tissue samples were processed in this study for the sole purpose of workflow validation. A large cohort of patients is under investigation to evaluate the concordance between CTCs and tumor tissues.

## Discussion

Enumeration and mutational profiling of CTCs are both important aspects of CTC research studies. As CTCs are rare and patient blood limited, it is highly desirable to use the same pooled CTC sample for both purposes. Fixation and staining are two steps of many current CTC enumeration workflows. The most popular fixative is PFA at various concentrations, with 4% being used most often. To use these fixed cells for genomic analysis, the proper DNA extraction method needs to be determined. In this study, several kits and protocols were tested and optimized on 4% PFA fixed HCT116 cells to identify the kit with the best performance and convenience. Among the four kits tested, the Qiagen GeneRead DNA FFPE kit workflow was more complicated than the others, with hazardous chemicals involved. The Arcturus® PicoPure® DNA Extraction kit had a simpler workflow but showed significant evaporation during incubation when using a 96 well-plate. The QIAamp DNA Micro kit required a simple procedure and could be used efficiently for both fresh and fixed cells. With further optimization on fixed cells using different incubation temperatures and times, a yield of about 80% was reached when incubating the cells at 60 °C overnight. In addition this kit showed similar performance on fixed, permeabilized, and stained cells and with low cell numbers (as low as 2 cells). This optimized DNA extraction protocol allowed the recovery of a sufficient DNA amount from fixed rare cells for genomic analyses and was successfully used in a study to detect KRAS, BRAF and PIK3CA mutations in CRC CTCs using Sanger sequencing.^8^ The ZR Genomic DNA™ Tissue MicroPrep kit showed equivalent performance for fixed cells, and could therefore be potentially used as a substitute for the Qiagen QIAamp Micro kit, but no further optimization has been performed in the presented study.

NGS, a key technological advance for the biosciences in the past decades, has been widely used in the cancer diagnostic field.^35^ Targeted DNA enrichment methods allow a high genome throughput at a feasible cost per sample. Many different platforms are currently available from different vendors. Choosing the right platform to satisfy the needs specific to CTC samples requires several considerations other than the performance metrics of read length, accuracy, and total sequence output. Minimum DNA input, gene panel size, gene cancer type specificity, and procedure simplicity also need to be considered. Several commercial targeted panels have been used by other groups on CTCs, e.g., Ion AmpliSeq™ Cancer Hotspot Panel v2 (Life Technologies), targeting 207 amplicons covering mutations from 50 oncogenes and tumor suppressor genes, was recently used to analyze mutations of single CTCs in metastatic breast cancer patients.^36^ This platform focuses on broad known cancer mutations but not unknown mutations. The TruSeq Amplicon Cancer Panel (Illumina) is a highly multiplexed targeted assay for detecting hundreds of mutational hotspots for 47 cancer specific genes and has been previously utilized on CTCs.^37^ This platform is very good at covering broad cancer types, including melanoma, colorectal, ovarian, and lung cancer but needs 150–250 ng of genomic DNA, which is well above the genomic resources obtainable from CTCs. GeneRead DNAseq Targeted Panels consist of multiplex PCR primer sets to amplify exonic regions of a thoroughly researched panel of cancer-focused genes and have been well used for rare tumor cells.^9,38^ The Human Colorectal Cancer Panel platform used in our study covers exonic regions of 38 colorectal cancer specific genes with the need of only 10–40 ng DNA. Thus, in this study, this platform was chosen to be further optimized on CTCs.

The GeneRead targeted NGS workflow performed well when applied to 40 ng DNA of both fresh and fixed HCT116 cells, with the detection of all expected mutations. The coverages were higher than 99% and the allele frequencies either at 100% for the homozygous mutations or close to 50% for heterozygous mutations. Application of this platform on gDNA from Roche human buffy coat gDNA, tumor tissue and FFPE blocks further demonstrates its good performance and confirms its broad usage on different tissue types. When used on lower amounts of DNA to evaluate its compatibility with CTC samples, 1.0 ng of DNA (about 200 cells) still performed well with all the expected mutations being detected at similar allele frequencies. However, inaccuracies including missed mutations or detection of additional mutations appeared when DNA input was decreased to 0.5 ng (~100 cells) or 0.2 ng (~40 cells). The lower the DNA amount, the higher the bias. It suggests that the platform could only be used for 200 cells or more. As CTC numbers collected from a cancer patient even with metastatic disease are rarely above 200 cells, an extra step of DNA amplification is needed.

WGA has been introduced to expand limited amounts of starting genomic material for NGS. Currently, three WGA strategies are widely used: degenerate-oligonucleotide-primed polymerase chain reaction (DOP-PCR), multiple displacement amplification (MDA), and a combination of displacement pre-amplification and PCR amplification. Hou et al. performed a thorough study to compare these different strategies^39^ and showed that QIAGEN REPLI-g Single Cell Kit had the highest mean genome coverage (8.84%) and significantly higher genome recovery sensitivity (~84%) compared to DOP-PCR (~6%) and MALBAC (~52%). This highlights that REPLI-g, using MDA technology, still is one of the best option among these WGA kits. In this study, we compared PCR based GenomePlex (WGA4) and MDA based REPLI-g. The WGA4 kit was first selected for testing its compatibility with fixed cells and a DNA input as low as 100 pg. Although the amplification seemed initially successful with a DNA amount going from 100 pg to 3000 ng, targeted NGS revealed a contamination of primers or linkers, and a coverage of only 48.2%. It is not clear whether this significant bias was due to the WGA4 amplification on fixed cells, or due to incompatibilities between WGA4 and the GeneRead panel. This was not further investigated in this study.

The REPLI-g Single Cell kit is especially designed to uniformly amplify genomic DNA from single cells (1 to <1000 cells) or purified genomic DNA with complete genome coverage. The MDA technology allows efficient DNA amplification and generates long DNA fragments (mainly >10 kb). However, this kit works better on fresh cells and was not verified on 4% PFA fixed cells by the manufacturer. This is why, in this study, we tested the REPLI-g on both fresh and fixed cell DNA, and obtained main DNA fragments greater than 12 kb for both. NGS results on amplified DNA showed a good coverage compared to non-amplified DNA for fresh cells (95.6 vs. 97.7%), but lower coverage for fixed cells (78.2 vs. 82.3%), suggesting that REPLI-g amplification followed by the Generead DNAseq targeted NGS workflow is more reliable for fresh than for fixed cells. Interestingly, the yield of amplification directly from fresh cells was much higher than from the cell DNA, with around 30 µg for ~15 cells. However, this step requires the collection of cells in a volume as little as 4 µl, which is challenging with current CTC platforms. We are working on approaches for CTC encapsulation in droplets to meet this volume requirement and to amplify DNA directly from CTCs, to obtain a high amount of intact DNA. This would potentially enable single cell NGS, whole exome sequencing and whole genome sequencing (WGS). Also, we noticed that the temperature of the cycler lid made a significant difference to the yield when performing the amplification step on a PCR cycler. A higher temperature could attenuate the Phi 29 polymerase’s efficiency, therefore we suggest using the lowest temperature that can be set. Besides the temperature, two protocols are available from Qiagen. The “standard protocol” is using an input of 2.5 µl DNA, while the “increased DNA volume protocol” is using 15 µl. As the output from column based DNA extraction is around 15–20 µl and all the output is needed to maximize CTC DNA recovery, the second approach was preferred and provided a DNA quantity sufficient to meet NGS requirement.

Purity is always a concern for studies on CTCs. The white blood cell background resulting from currently available CTC enrichment platforms is typically high, and further CTC purification to achieve sufficient purity for NGS platforms is usually necessary. Single CTC picking and sequencing is preferable but manual microscope manipulation of single cells is time consuming and cumbersome.^40,41^ Platforms for single cell manipulation have been developed but at a higher cost per sample. To perform NGS on pooled cells (CTCs and blood cells), first a good CTC enrichment platform with high capture efficiency and purity is needed. A second requirement is to collect the WBCs from the same patient as a germline control, to eliminate the DNA mutational background of blood cells from the CTC signal of interest.

Vortex technology, a label-free microfluidic-based device (Vortex Biosciences Inc.) has demonstrated its ability to isolate and concentrate CTCs at high purity from whole blood samples.^21,22^ In this study, blood samples from three CRC patients and two healthy donors were processed through Vortex microfluidic device. Large quantities of CTCs were captured in two of three patient samples, with many of them being clusters. The cells collected were analyzed for mutation profiling using the tested and optimized workflow, and somatic mutations were called in CTCs after removal of germline mutations from WBC controls. This confirms the possibility of using pooled CTCs for targeted NGS. Interestingly, most of the CTCs isolated from these two patients did not show any CK expression but were only vimentin and N-Cadherin positive. Vimentin is an important marker of epithelial mesenchymal transition (EMT), a process characterized by upregulation of mesenchymal markers (vimentin, fibronectin, and N-cadherin). The dissemination of EMT positive cells is an important feature of cancer metastatic progression and these cells, negative for epithelial markers (CK, EpCAM), would have been missed by epithelial marker based approaches. As CTC capture with Vortex technology is a label-free and contact-free process, isolated CTCs remain unbiased by molecular characteristics and are unaltered by physical filters, labels or reagents, which opens up new opportunities for researchers.

A limitation of our study is that we did not use specific markers to identify other circulating cells in cancer patients, such as circulating endothelial cells^42^ and circulating cancer-associated fibroblasts.^43^ As multiplex marker systems become more commonly used for CTC phenotyping, the roles of these cells and any mutations they may carry will become better understood.

In summary, this study offers broad methodological instructions on how to perform NGS on tumor tissue and rare cells such as CTCs, fixed or fresh. GeneRead DNAseq targeted sequencing. It performs well when sufficient DNA is obtained for example from tissue, cell lines or, when coupling with REPLI-g for fresh rare cells such as CTCs collected from Vortex technology. Here, the high purity of Vortex enriched CTCs and the use of a WBC germline control made NGS on pooled cells possible. Overall this study offers researchers a possible workflow for CTC genomic analysis using purified CTCs and targeted sequencing. Using this optimized workflow, future concordance studies may be done to compare CTCs, ctDNA, and primary and metastatic tumor tissue from the same patient. In addition to aiding the clinical integration of these technologies, this may help further elucidate cancer heterogeneity and the metastatic process.

## Materials and methods

### Cell culture and preparation

The human colorectal cancer cell line HCT116 (ATCC, American Type Culture Collection; CCL-247, tested for mycoplasma contamination) was used as a model for testing and optimization of all protocols. HCT116 cells were grown at 37 °C and 5% CO_2_, in McCoy’s 5a medium (Gibco), supplemented with 10% fetal bovine serum (HyClone) and 1% Penicillin-Streptomycin (Corning). *Preparation of fixed cells:* after centrifugation (1000 r.p.m., 5 min), HCT116 cells were re-suspended in 4% Paraformaldehyde (PFA, Electron Microscopy Sciences) for 10 min, washed three times with PBS, re-suspended in PBS, and counted by using a hemocytometer before use. *Preparation of permeabilized cells:* following fixation and wash, cells were permeabilized at room temperature for 7 min using a 1:1 mix of 0.4% Triton-X and 10% goat serum, then washed three times with PBS. Until the DNA extraction was performed, fresh cells were stored at −80 °C, while fixed and fixed+permeabilized cells were stored at 4 °C.

To test DNA extraction protocols, fresh or fixed cells were collected in either 1.5 ml micro-centrifuge tubes (Axygen, Maxymum Recovery) or in 96 well-plates (Costar). Fifty microgram of the same cell mix (serially diluted to obtain a certain density) was seeded simultaneously into a micro-centrifuge tube and a well for cell counting. The cells in the well-plate were then imaged using a microscope (Axio Observer Z1Zeiss), and manually enumerated in order to calculate the DNA yield accurately. Such method enables the DNA extraction from a very low number of cells (as low as five cells) while knowing exactly how many cells are being processed from the well. A stock of several cell aliquots was prepared at once to eliminate potential variability while testing different kits and different protocols at different times.

### DNA extraction

Different kits and protocols were used to extract DNA from cell lines, tissues and blood. *To extract DNA from fresh cells*, the QIAamp Micro Kit (Qiagen) was used following the standard cell protocol from Qiagen. *To extract DNA from fixed cells*, the QIAamp DNA Micro kit (Qiagen), GeneRead DNA FFPE kit (Qiagen), ZR Genomic DNA™ Tissue MicroPrep kit (Zymo) and Arcturus® PicoPure® DNA Extraction kit (Life technologies) were tested following the manufacturer’s standard protocols. After comparison of the different kits, due to good results, the QIAamp DNA Micro kit was selected with a modified version of the manufacturer’s protocol: Briefly, the well-plates with the cell suspensions were centrifuged at 2000 rpm for 2 min and PBS was cautiously removed. Tissue lysis buffer ATL and proteinase K were added to the well and incubated overnight at 60 °C. Then, the lysate was transferred from the well-plate to a microcentrifuge tube. Lysis buffer AL was then added to continue the lysis step. The whole lysate was then loaded into the provided column. After being washed with buffers AW1 and AW2, the bound DNA was eluted in 25 µl water. *To extract DNA from tissue*, the QIAamp DNA Micro Kit was used following the tissue protocol for frozen metastatic liver tissues (10 mg), whereas for FFPE samples of primary cancer tissues (5 μm per section, 2 sections), the GeneRead DNA FFPE Kit was used following the standard protocol from Qiagen. *To extract DNA from WBCs*, the QIAamp DNA Micro Kit was used on 100 µl of whole blood following the blood protocol.

### DNA quantification

When DNA yield was expected to be high, such as for the DNA extracted from tissue samples, DNA was quantified using Qubit™ 3.0 Fluorometer (Thermo Fisher) with the Qubit® dsDNA HS Assay Kit (Thermo Fisher). To quantify the DNA extracted from rare cells, a more sensitive and accurate method was needed. Therefore, an absolute quantitative qPCR was performed using the 7500 Fast Real-Time PCR system (Applied Biosystems®) and human long interspersed nuclear element-1 as the target gene (Forward primer: 5′-TCACTCAAAGCCGCTCAACTAC-3′, and Reverse primer: 5′-TCTGCCTTCATTTCGTTATGTACC-3′).^44^ Various dilutions of normal human blood (buffy coat) genomic DNA (Roche Diagnostics Corporation) purified from human blood (buffy coat) were incorporated in each experiment to serve as standards.

### Whole genome amplification

WGA was performed on the extracted DNA by two different technologies, GenomePlex® Single Cell Whole Genome Amplification Kit (WGA4) from Sigma and REPLI-g Single Cell Kit from Qiagen.

*For GenomePlex WGA4 kit:* 10 μl of DNA was fragmented for 4 min at 99 °C after adding 1 μl of 10× Single Cell Lysis and Fragmentation Buffer. Then, a set of random primers coupled with common adapters was linked to the fragmented DNA template at 16 °C for 20 min, 24 °C for 20 min, 37 °C for 20 min, 75 °C for 5 min to generate the library. PCR was then performed to amplify the library with an initial denaturation at 95 °C for 3 min, 25 cycles of 94 °C for 30 s and 65 °C for 5 min. The PCR amplified DNA was then purified using the Qiagen QIAquick PCR Purification Kit.

*For REPLI-g Single Cell Kit:* The standard protocol “Whole genome amplification from genomic DNA using the REPLI-g® Single Cell Kit” from Qiagen, as well as the protocol “increased volume” were both used. Briefly, 15 µl template DNA was loaded into a 0.2 ml PCR tube. Two microgram of denaturation buffer DLB was added to the DNA and the mixture was incubated at room temperature for 3 min. Later, 3 µl of Stop Solution was added to stop the lysis reaction. A WGA master mix containing 29 μl REPLI-g sc Reaction Buffer and 2 μl of REPLI-g sc DNA Polymerase was added to the cell lysate followed by the isothermal amplification at 30 °C for 8 h and inactivation at 65 °C for 3 min.

The amplified DNA was then purified using Agencourt® AMPure® XP magnetic beads. For both methods, the concentration of purified WGA DNA was measured by Qubit and DNA yield was calculated. The purified WGA DNA was also loaded onto 1% E-Gel electrophoresis system (Thermo Fisher) to check the DNA size and quality.

### Targeted panel multiplexed PCR and NGS

For gene mutation analysis, GeneRead DNAseq Human CRC targeted panel v2 (NGHS-002X, Qiagen) based MiSeq NGS was selected and verified. The panel contains 1954 primer sets covering all exons of the following 38 colorectal cancer related genes (Supplementary Figure 1): *ACVR1B, AKT1, APC, ATM, ATP6V0D2, BAX, BRAF, CASP8 (FLICE), CDC27, CTNNB1, DCC, DMD, EP300, ERBB2 (HER2), FBXW7, FZD3, GPC6, KRAS, MAP2K4 (JNKK1), MAP7, MIER3, MLH1, MSH2, MSH3, MSH6, MYO1B, NRAS, PIK3CA (p110α), PIK3R1, PTPN12, SLC9A9, SMAD2, SMAD4, TCERG1, TCF7L2, TGFBR2, TP53, WBSCR17*.

#### Multiplexed PCR targeted enrichment

Forty microgram of genomic DNA was combined with primer mix and PCR reagent, and amplified in four separate PCR reactions (10 ng/pool). PCR was performed with Veriti Thermal Cycler (Applied Biosystems) and the following conditions: 15 min at 95 °C for denaturation, 18 cycles of amplification at 95 °C for 15 s, 60 °C for 4 min, 72 °C for 10 min for extension, and a final maintenance at 4 °C. The amplified PCR products were then pooled, purified with the Agencourt AMPure XP beads (Beckman Coulter) and subjected to library preparation including adapter ligation, purification, and size selection using the TruSeq library prep kit (Illumina). A final PCR was performed to further amplify adapter-carrying fragments. Amplified amplicons were purified with AMPure Beads (Beckman Coulter) and their concentration was determined according to the manufacturer’s protocol.

#### MiSeq sequencing

The DNA library of each sample was diluted to a final concentration of 2 nM. Libraries from 15 samples were mixed to a sample library pool. 0.2 M NaOH was added to the library pool to denature the DNA. A PhiX spike in control (Illumina) was denaturized in the same manner and both the sample library pool and the PhiX control were then diluted to 10 pM. 1% of PhiX was added to the sample pool and 600 µl of this final solution was loaded into the MiSeq cartridge (Illumina) following the manufacturer’s instructions. Sequencing was performed (SeqMatic, Fremont, CA) on a MiSeq sequencer using v2 chemistry (Illumina).

### Data analysis and interpretation

Sequencing FASTQ files generated by the MiSeq Reporter program (Illumina Inc.) were uploaded into QIAGEN NGS Data Analysis Web Portal for analysis. This cloud-based software automatically performs all the steps necessary to generate a DNA sequence variant report from the NGS data. The disease-causing variants were further analyzed using Ingenuity® Variant Analysis™ software (www.qiagen.com/ingenuity), with the following filter cascade: Variants→Confidence→Common Variants→Predicted Deleterious→Genetic Analysis→Cancer Driven Variants. The “predicted deleterious” filtration keeps only the variants experimentally observed to be associated with a pathogenic or likely pathogenic phenotype according to computed American College of Medical Genetics Guidelines classification. The filtration of “Cancer Driven Variants” keeps only variants that are found in cancer-associated events, cancer therapeutic targets or published cancer literature. For our variant analyses, we specifically selected colorectal cancer as the entry for cancer type.

To analyze the gene coverage, the FASTQ files were first aligned to the human genome reference hg19 using BWA MEM. Then, DepthOfCoverage function from Genome Analysis Toolkit (GATK)^45^ and the coverage statistics for all locations of genome were computed (command: *java -Xmx5G -jar GenomeAnalysisTK.jar -L genepanel.bed -o OUTPUT.txt -I INPUT.BAM -T DepthOfCoverage --summaryCoverageThreshold 10 -R hg19.fasta)*. The coverage over individual genes was computed by in-house programs. Only bases with a minimum base quality score of Q30 were considered. The graphs show the coverage +/− one-standard deviation for genes of interest. The percentage of bases covered by at least 10 reads (%_bases_above_10) was also calculated using data obtained from DepthOfCoverage and in-house scripts.

### Donor recruitment and sample collection

All donors were recruited at Stanford University Hospital, according to a clinical study protocol approved by the Institutional Review Board (Stanford IRB # 5630) (Supplementary Table 1). Prior to being enrolled into the study, all donors provided written informed consent. In a first study (Study I), tissue specimens—including three primary tumor samples (FFPE), 14 liver metastatic tumor samples (fresh frozen tissue) and lung metastatic tumor sample (FFPE)–were collected from a cohort of 14 patients with stage IV colorectal cancer with surgically resectable metastatic disease to the liver. In order to test our established workflow for NGS, in a second study (Study II), tissue specimens as well as blood samples were collected from three patients, also with stage IV colorectal cancer with surgically resectable metastatic disease to the liver. Metastatic tumor tissue (0.5–1.0 g) was collected during surgery, transported on ice and stored at −80 °C until further processing. Blood samples were collected prior to surgery, stored at room temperature and processed within 8 h of collection. In parallel, blood specimens were collected from two age-matched donors without history of malignant disease.

### CTC isolation, immunostaining and enumeration

Blood samples were collected into two EDTA tubes. One tube of blood (8 ml) was processed with Vortex platform to collect and enumerate CTCs and another one was processed to collect CTCs for genomic analysis. Additionally, 100 µl of whole blood from this same tube was used as a germline control. Flow was driven through Vortex plastic device by the use of two syringe pumps (Harvard Apparatus), one for the blood sample solution and one for the wash buffer. The fluidic processing includes priming, capture, wash and release steps as previously described.^8,21,22^ Cells captured in the vortices were released either into a 96 well-plate for downstream fixation, immunostaining, imaging, and enumeration, or into a 1.5 ml micro-centrifuge tube for DNA extraction, WGA and further sequencing. For immunostaining, isolated cells were fixed with 4% PFA for 10 min, blocked with 5% Goat Serum (Invitrogen) for 30 min, and then immunostained with DAPI (Life Technologies), anti CK-fluorescein isocyanate (CK-FITC, CAM 5.2, BD Biosciences, Ref#347653, CK-FITC, CK3-6H5, MACS Miltenyi, Ref#130-080-101, PanCK-AF488, AE1/AE3, eBioscience, Ref#53–9003-82), anti-Vimentin (Alexa Fluor® 647, CloneV9, Abcam, Ref#195878), anti N-cadherin (Alexa Fluor® 647, EPR1791-4, Abcam, Ref#195186) and anti CD45-phycoerythrin (CD45-PE, Clone HI30, BD Biosciences, Ref#555483) antibodies. Note that Vimentin and N-cadherin were labeled with the same Alexa Fluor so they could be detected on the same channel channel. Using an Axio Observer Z1 microscope (Zeiss), stitched images of stained cells were acquired, and cells were manually enumerated by two different persons following a classification criteria developed at Vortex. The cells were categorized into three large groups, namely CTCs, WBCs or debris. CK+/CD45-/DAPI+ and VIM+/N-Cad+/CD45-/DAPI+ cells were identified as CTCs, while CK-/CD45+/DAPI+, VIM-/N-Cad-/CD45+/DAPI+ and VIM+/N-Cad+/CD45+/DAPI+ were identified as WBCs. Cells with other staining patterns (DAPI only) were classified as CTCs or WBCs according to their morphological features following standard methods used in cytopathology.^8,21^ The number of CTC-like cells (faint CK, faint VIM and N-Cad) and leukocytes was documented and included in the CTCs per milliliter of whole blood calculation.

### Data availability statement

The authors declare that all the data supporting the findings of this study are available within the paper and its supplementary information files.

## Electronic supplementary material

Supplementary Material
