# Supplementary material for: Workflow optimization of whole genome amplification and targeted panel sequencing for CTC mutation detection
Source: NPJ Genom Med. 2017 Nov 1;2:34. doi: 10.1038/s41525-017-0034-3 (PMC5677973; doi:10.1038/s41525-017-0034-3)
Supplement: Supplementary file 1 — Supplementary Material [file 41525_2017_34_MOESM1_ESM.docx]

**Workflow Optimization of Whole Genome Amplification and Targeted Panel Sequencing for CTC Mutation detection**

**AUTHORS**

Haiyan E. Liu^1^, Melanie Triboulet^2^, Amin Zia^3^, Meghah Vuppalapaty^1^, Evelyn Kidess-Sigal^2,4^, John Coller^5^, Vanita S Natu^5^, Vida Shokoohi^5^, James Che^1^, Corinne Renier^1^, Natalie H. Chan^2^, Violet R. Hanft^2^, Stefanie S. Jeffrey^2^*, Elodie Sollier-Christen^1^*.

* These authors contributed equally and both are corresponding authors.

**SUPPLEMENTARY FILES**


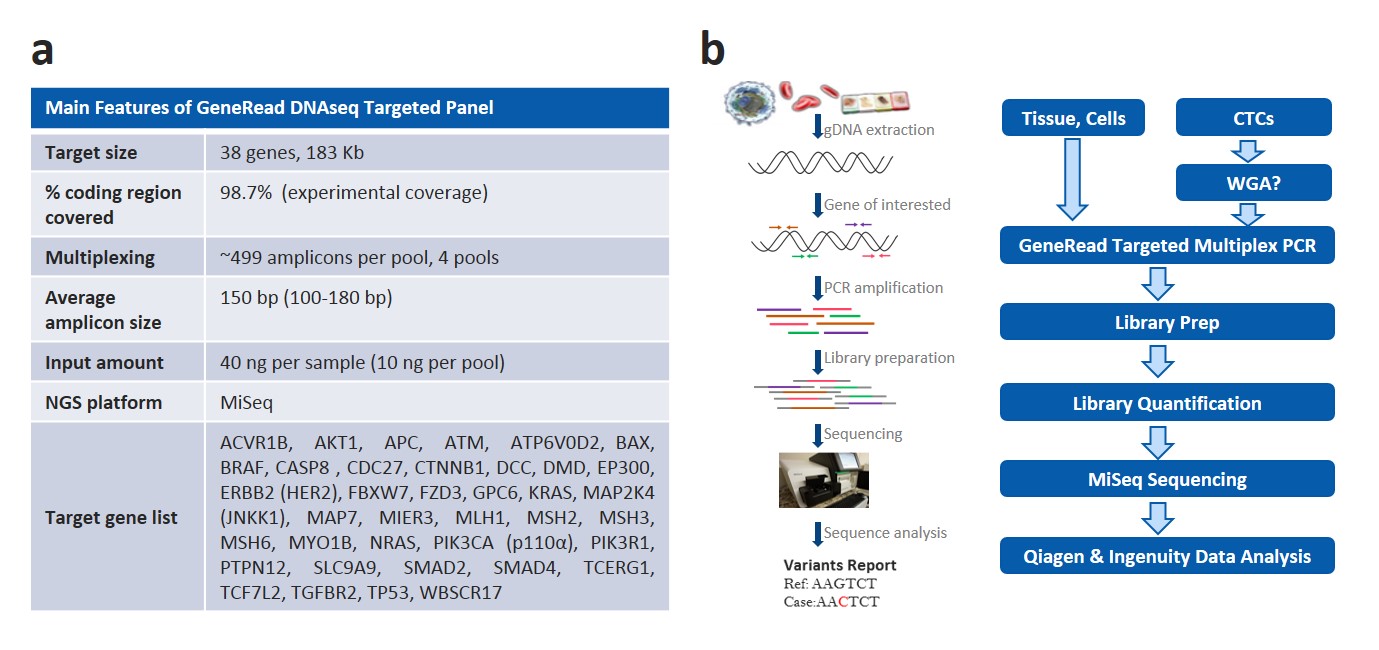


**Supplementary Figure 1: GeneRead DNAseq Targeted Panel and Workflow.** (a) Key features of the Human Colorectal Cancer GeneRead DNAseq Targeted Panel. (b) Description of the sample-to-insight workflow for DNA sequencing with GeneRead DNAseq Targeted Panel.

**
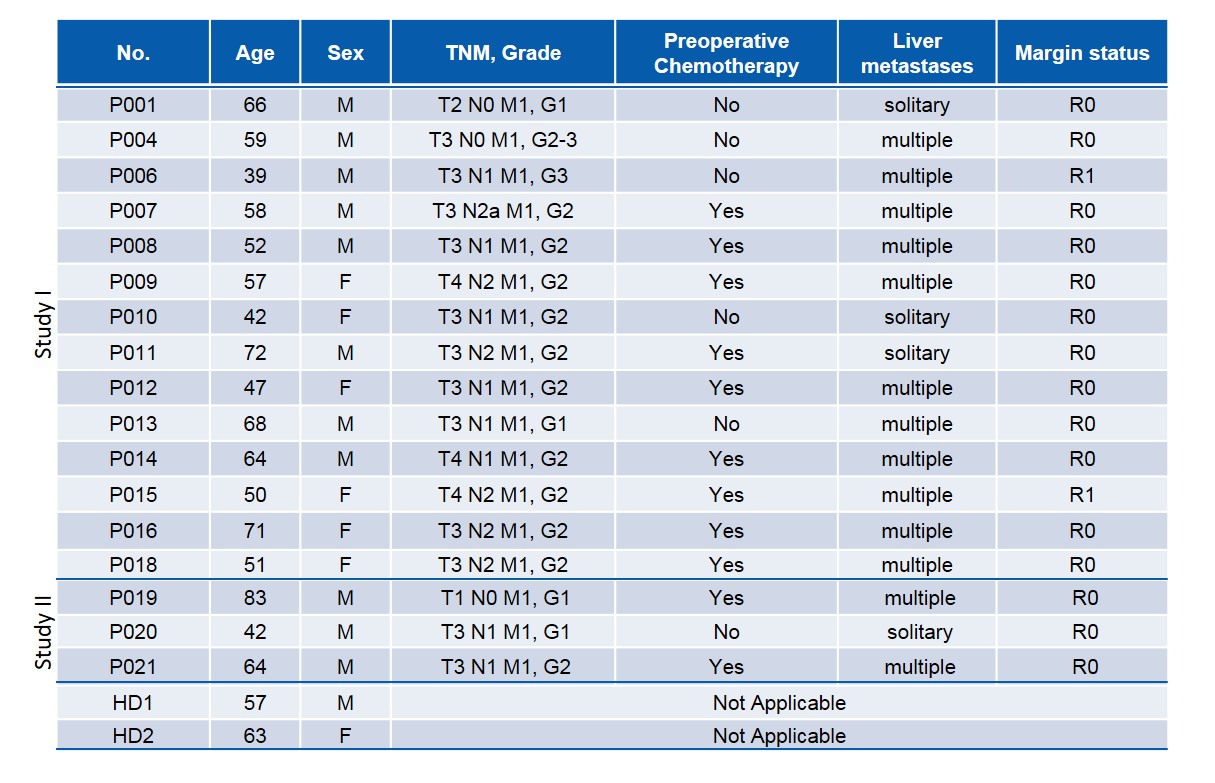
**

**Supplementary Table 1: Characteristics of Patients and Healthy Donors*.*** Resection margin status R0=No residual tumor microscopically; R1=microscopically residual tumor.


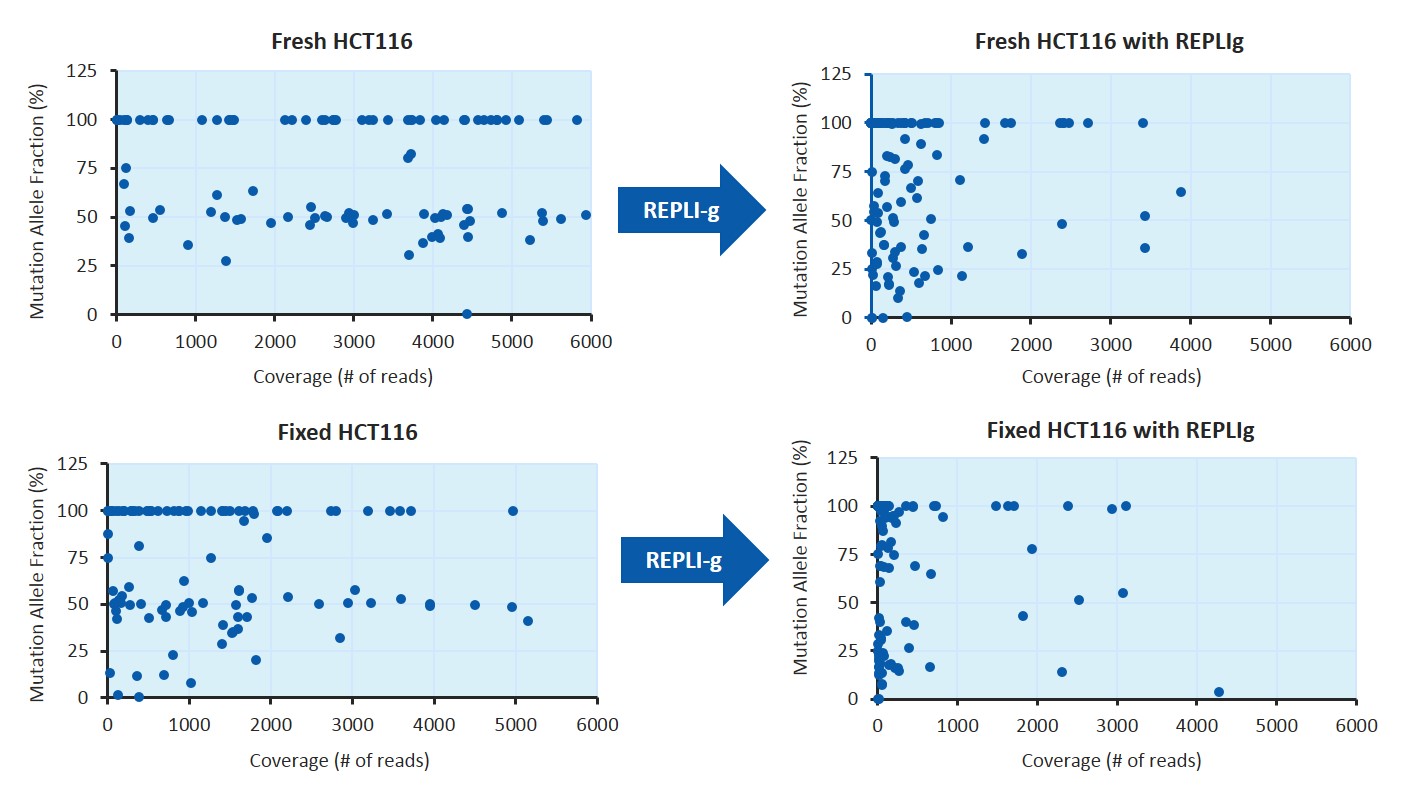


**Supplementary Figure 2:** MAF alteration after WGA. MAF of mutation sites between WGA amplified and non- amplified samples were compared. The allele frequencies of the SNP in fresh HCT116 cells (top left) are around 100% and 50%, which represents both homozygous and heterozygous SNP frequencies. The same trend was observed in the fixed HCT116 cells (bottom left). In fresh samples, after REPLI-g WGA (top right), most homozygous SNPs underwent an even amplification with still 100% of MAF, while the MAF of the heterozygous SNP varied significantly, emphasizing the strand bias of WGA. This strand bias was much more severe when REPLI-g WGA was applied on fixed cells (bottom right).
